# Phocine Distemper in German Seals, 2002

**DOI:** 10.3201/eid1004.030591

**Published:** 2004-04

**Authors:** Gundi Müller, Peter Wohlsein, Andreas Beineke, Ludwig Haas, Irene Greiser-Wilke, Ursula Siebert, Sonja Fonfara, Timm Harder, Michael Stede, Achim.D. Gruber, Wolfgang Baumgärtner

**Affiliations:** * School of Veterinary Medicine Hannover, Hannover, Germany; † Christian-Albrechts-University at Kiel, Büsum, Germany; ‡ Food, Veterinary and Environmental Diagnostic Laboratory of Schleswig-Holstein, Neumünster, Germany; § State Veterinary Investigation Centre for Fish, Cuxhaven, Germany

**Keywords:** harbor seal, phocine distemper virus, germany, RT-PCR, immunohistochemistry, serology

## Abstract

Approximately 21,700 seals died during a morbillivirus epidemic in northwestern Europe in 2002. Phocine distemper virus 1 was isolated from seals in German waters. The sequence of the P gene showed 97% identity with the Dutch virus isolated in 1988. There was 100% identity with the Dutch isolate from 2002 and a single nucleotide mismatch with the Danish isolate.

In the past, fatal morbillivirus infections have been reported in various cetacean and seal species. In pinnipeds, the disease has been described in crabeater seals (*Phoca carcinophagus*) from the Antarctic (*1*), Baikal seals (*P. siberica* [*2*]), monk seals (*Monachus monachus*; [*3*]), Caspian seals (*P. caspica* [*4*]), and harbor seals (*P. vitulina*) from the North and Baltic Sea (*5*). Phocine and canine distemper viruses (CDV) were isolated as causative agents in different epidemics of seals. Phocine distemper virus 1 (PDV-1) and CDV represent two distinct but antigenetically and genetically related morbilliviruses (*5*). An increased number of deaths in the Danish seal population was noticed in May 2002, starting at the Kattegat Isle of Anholt. PDV-1 was isolated as the causative agent (*6*), and the disease spread to Sweden and Norway in the following month. A second outbreak was observed mid-June in the Netherlands. Subsequently, the disease spread to Germany and Denmark in an eastern direction, and to Belgium, France, Great Britain, and Ireland to the West. In Germany, approximately 7,500 harbor seals died during the epidemic (*7*). We present morphologic, virologic, and serologic findings in affected seals from German waters.

## The Study

**Figure 1:**
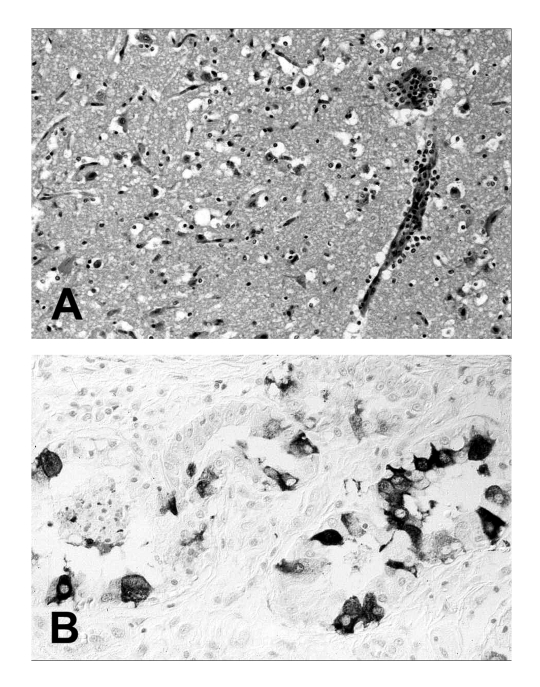
Tissue lesions from a harbor seal (*Phoca vitulina*) with phocine distemper virus infection. (A) Cerebral cortex with nonsuppurative encephalitis. Hematoxylin and eosin staining. (B) Immunohistochemical labeling of morbilliviral antigen in glandular epithelial cells of the lung. Avidin-biotin-peroxidase technique with Papanicolaou’s hematoxylin counterstain.

Necropsies of 95 harbor seals (*P. vitulina*) collected from July to December 2002 showed a moderate-to-severe pulmonary alveolar and interstitial emphysema and alveolar edema as the predominant findings. Additional lesions included mediastinal emphysema, gradually variable suppurative bronchopneumonia, and catarrhalic enteritis. Histologic lesions consisted of interstitial pneumonia with multinucleated syncytial cells and a moderate-to-severe lymphocytic depletion in the lymphoid tissues. Single animals had an acute, focal, nonsuppurative encephalitis (Figure 1A). In addition, neuronal necrosis and mild gliosis were observed. Cytoplasmic and nuclear acidophilic inclusion bodies were detected in respiratory epithelial cells, gastric surface mucous and chief cells, intestinal crypt epithelial cells, and hepatic and pancreatic duct epithelial cells. In the urogenital tract, inclusion bodies were observed in endometrial, vaginal, and epididymal epithelial cells as well as epithelial cells of the renal pelvis and urinary bladder. Occasionally, inclusion bodies were present in neuronal and glial cells of the central nervous system.

Immunohistochemical analyses were performed by using a cross-reacting murine monoclonal antibody specific for the morbillivirus nucleoprotein. Morbillivirus antigen was demonstrated in 39 (45%) of the 86 cases. Morbillivirus antigen was detected in lung, trachea, stomach, intestine, liver, pancreas, kidneys, urinary bladder, female genital mucosa, and epididymal tubules (Figure 1B). In the lymphoid tissues, variable numbers of lymphocytes and macrophages of the follicular and parafollicular areas were positive. In affected areas of the brain, neurons and glial cells contained morbillivirus antigen in the nuclei and cytoplasm.

Screening for morbillivirus-specific nucleic acid in tissue samples from lung, spleen, and lymph nodes as well as in blood samples from 85 seals was performed by reverse transcription–polymerase chain reaction (RT-PCR). For this procedure, universal morbillivirus primers based on the conserved sequence of a 457-bp fragment of the phosphoprotein gene (*8*,*6*) were used. PDV-specific RNA was detected in 46 (54%) of the 85 seals from German waters affected from July onward. Both PDV-specific RNA and morbillivirus antigen were detected in 33 (43%) of 77 animals. Seals with no detectable morbillivirus antigen or nucleic acid had pneumonia and endoparasitosis of varying degrees of severity or died of undetermined causes.

**Figure 2:**
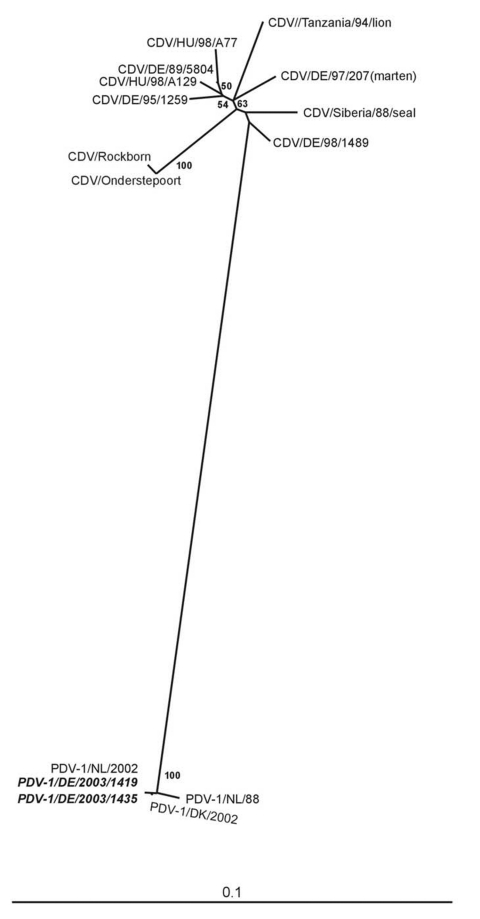
Unrooted neighbor-joining phylogenetic tree constructed by using 369 nt from the gene coding for the morbillivirus P protein. Alignments were calculated with CLUSTAL X (Version 1.8). Bootstrapping (values indicated in %) was performed with 1,000 replicates. TREEVIEW (Version 1.6.5) was used for the graphic display of the tree. The canine distemper virus (CDV) sequences included were from vaccine strains Rockborn (AF181446) and Onderstepoort (AF378705), Siberian seal (AF259551), lion (U76708); CDV isolates originating from dogs 5804/89 (AJ582384), A129/98 (AJ582385), A77/98 (AJ582386), 1489/98 (AJ582387), and 1259/95 (AJ582388); and 207/97 (AJ582389), which was isolated from a marten. Phocine distemper virus (PDV) isolates were PDV-1/NL/88 (AF525289); PDV-1/DK/2002 (AF525287); and PDV-1/NL/2002 (AF525288). The German PDV isolates 1435 and 1419 are in a discrete cluster with a Dutch and with a Danish seal PDV isolate from 1988 and 2002, respectively. Bar, nucleotide substitutions per site.

Sequence analysis of the RT-PCR product showed an identity of 97% compared to the Dutch isolate of 1988. The German isolate was 100% identical with the PDV isolate from the Netherlands and differed in 1 nt from the Danish isolate (*6*) (not shown). Phylogenetic analysis showed that the phocine isolates from the two epidemics in European waters formed a discrete cluster, separated from the CDV isolates, including those from lion and Siberan seal (Figure 2).

Neutralization assays using the CDV strain Onderstepoort were performed to determine the titers of serum samples from 187 harbor seals from German waters, collected from 1996 until the outbreak of the epidemic in 2002 (*9*,*10*). Because of the cytotoxicity of some serum samples, only titers of >10 were considered positive. No neutralizing antibodies were found in 164 (88%) of 187 serum samples. Titers from 22 (12%) of the 187 animals ranged from 14 to 240 (mean 50.5, ± 52.6 standard deviation). One animal had a titer of 480.

## Conclusions

The morphologic and immunohistochemical findings in harbor seals from German waters during the recent morbillivirus epidemic in northwestern Europe closely resembled those observed in 1988 (*11*–*13*,*5*) and confirmed the epithelio-, lympho- and neurotropism of the PDV. The distribution of the viral antigen indicates that the respiratory tract was the primary route of morbillivirus infection. The virus-induced marked lymphoid depletion may have allowed secondary bacterial infections. In contrast to reports about European harbor seals from 1988, no demyelination was detected in seals from German waters in 2002 (*5*). Whether this finding represents a distinct feature of the 2002 epidemic or is a result of the small number of investigated animals remains unclear. Seals that died during the morbillivirus epidemic with no detectable viral antigen or nucleic acid may have cleared the virus but still have virus-induced immunosuppression, which could result in fatal secondary bacterial or parasitic infections. Furthermore, poor preservation of some carcasses may have caused false-negative results. The RNA sequences of the recent virus isolates showed a virus population along the German coast during this epidemic that was almost identical to the isolates from the Netherlands and Denmark in 2002 and that had a high identity to the isolate from 1988 (*6*). Protective morbillivirus-specific antibody titers were detectable in only a few seals from German waters before the outbreak in 2002, suggesting a high susceptibility for morbillivirus infection in this naive population.

During the morbillivirus epidemic in 1988, approximately 65% of the Dutch, Danish, and German Wadden Sea seal population died (*7*). The death rate in 2002 is estimated at approximately 51% on the basis of the number of dead seals and the count of the Wadden Sea seal population in 2003 (*14*). The lower death rate in 2002 may have been influenced by different factors, such as decreased social contacts at the beginning of the epidemic during the late breeding season. In addition, genetic selection of a less susceptible population originating from the survivors of the 1988 outbreak might have resulted in a lower number of deaths during the second epidemic. It remains unclear why both outbreaks started at the Danish Kattegat isle of Anholt. In the past, migrating Arctic seal species, such as harp seals from Greenland, have been suspected as carriers that introduced a morbillivirus into an immunologically naive population (*15*). This species may have served as a reservoir that maintains the circulation of PDV.

Several epizootics of infectious diseases in marine mammals with increases in air temperature were observed, indicating that environmental influences may have also resulted in the emergence of new epidemics (*16*). Further studies are needed to determine whether alterations in migration patterns of Arctic seal species caused by changes in climatic conditions are responsible for the two PDV epidemics in northwestern Europe.

## References

[1] Bengtson JL, Boveng P, Franzen U, Have P, Heide-Jorgensen MP, Harkonen TL. Antibodies to canine distemper virus in Antarctic seals. Mar Mamm Sci. 1991;7:85–7. 10.1111/j.1748-7692.1991.tb00553.x

[2] Grachev MA, Kumarev VP, Mamaev LV, Zorin VL, Baranova LV, Denikina NN, Distemper in Baikal seals. Nature. 1989;338:209–10. 10.1038/338209a0 2922047

[3] Van de Bilt MWG, Vedder EJ, Martina BEE, Sidi BA, Jiddou AB, Barham MEO, Morbilliviruses in Mediterranean monk seals. Vet Microbiol. 1999;69:19–21. 10.1016/S0378-1135(99)00082-6 10515264

[4] Kennedy S, Kuiken T, Jepson PD, Deaville R, Forsyth M, Barrett T, Mass die-off of Caspian seals caused by canine distemper virus. Emerg Infect Dis. 2000;6:637–9. 10.3201/eid0606.000613 11076723 PMC2640919

[5] Kennedy S. A review of the 1988 European seal epizootic. Vet Rec. 1990;▪▪▪:563–7. 2288059

[6] Jensen T, van de Bildt M, Dietz HH, Andersen TH, Hammer AS, Kuiken T, Another phocine distemper outbreak in Europe. Science. 2002;297:209. 10.1126/science.1075343 12114617

[7] Bericht des Ministers für Umwelt. Natur und Forsten an den Umweltausschuss des Landtages. Verlauf der Seehundstaupeepidemie im schleswig-holsteinischen Wattenmeer im Jahr 2002. Stand: Jan 2003. Available from: http://www.wattenmeer-nationalpark.de/seehundstaupe.pdf (in German).

[8] Barrett T, Visser IKG, Mamaev L, Goatley L, van Bressem MF, Osterhaus ADME. Dolphin and porpoise morbilliviruses are genetically distinct from phocine distemper virus. Virology. 1993;193:1010–2. 10.1006/viro.1993.1217 8460473

[9] Frisk AL, König M, Moritz A, Baumgärtner W. Detection of canine distemper virus nucleoprotein RNA by reverse transcription-PCR using serum, whole blood, and cerebrospinal fluid from dogs with distemper. J Clin Microbiol. 1999;37:3634–43. 10523566 10.1128/jcm.37.11.3634-3643.1999 PMC85712

[10] Müller G, Siebert U, Wünschmann A, Baumgärtner W. Immunohistological and serological investigation of morbillivirus infection in harbour porpoises (*Phocoena phocoena*) from the Baltic and North Sea. Vet Microbiol. 2000;75:17–25. 10.1016/S0378-1135(00)00209-1 10865149

[11] Bergman A, Järplid B, Svensson BM. Pathological findings indicative of distemper in European seals. Vet Microbiol. 1990;23:331–41. 10.1016/0378-1135(90)90164-Q 2402876

[12] Heide-Jorgensen MP, Harkonen T, Dietz R, Thompson PM. Retrospective of the 1988 European seal epizootic. Dis Aquat Organ. 1992;13:37–62. 10.3354/dao013037

[13] Heje NI, Henriksen P, Aalbaek B. The seal death in Danish waters 1988. I. Pathological studies. Acta Vet Scand. 1991;32:205–10. 1803933 10.1186/BF03546982 PMC8127878

[14] Reijnders PJH, Brasseur S, Abt KF, Siebert U, Stede M, Tougaard S. The harbour seal population in the Wadden Sea as revealed by the aerial surveys in 2003. Wadden Sea Newsletter. 2003;2:11–2.

[15] Dietz R, Hansen CT, Have P, Heide-Jorgensen MP. Clue to seal epizootic. Nature. 1989;338:627. 10.1038/338627a0 2704417

[16] Lavigne DM, Schmitz OJ. Global warming and increasing population densities: a prescription for seal plagues. Mar Pollut Bull. 1990;21:280–4. 10.1016/0025-326X(90)90590-5

